# Misled by the Air: Pneumocephalus

**DOI:** 10.7759/cureus.2480

**Published:** 2018-04-14

**Authors:** Koin Lon Shum, Yuyang Tan

**Affiliations:** 1 Department of Internal Medicine, Singapore General Hospital

**Keywords:** pneumocephalus, air, skull, stroke

## Abstract

Pneumocephalus, the presence of air within the cranium, commonly suggests a breach in the meningeal layer or an intracranial infection by a gas-producing organism. Trauma is the most common cause of pneumocephalus, followed by cranial surgery. Other causes include infection and intracranial neoplasm.

An 87-year-old man was conveyed to the emergency department after being found to be drowsy by his helper. He was noted to have a new onset right-sided hemiparesis. Past medical history was significant for hypertension, stage 5 chronic kidney disease, cerebrovascular accident, pacemaker insertion for sick sinus syndrome, transurethral resection of the prostate for benign prostatic hyperplasia, and pulmonary tuberculosis. Computed tomography (CT) of the brain revealed pneumocephalus with air within the dural venous sinuses. A facial bone CT that was performed to look for a fracture demonstrated a minimally displaced fracture of the lateral wall of the right maxillary sinus. There was an acute left middle cerebral artery territory infarct with a hemorrhagic conversion. Despite medical treatment, the patient demised one month after the initial presentation.

Pneumocephalus is an uncommon finding, even in trauma. In the event that the clinical presentation cannot be explained by the mere presence of air within the cranium, another diagnosis ought to be sought. The delay in finding an alternative diagnosis and its management can be disastrous or even fatal.

## Introduction

Pneumocephalus is defined as air within the cranium. It is an uncommon finding and suggests a breach in the meningeal layer or an intracranial infection by a gas-producing organism. Trauma remains the most common cause of pneumocephalus, followed by cranial surgery, intracranial neoplasm, and infection [1]. We report a patient with idiopathic pneumocephalus, which could not explain the initial presenting complaint and was later diagnosed to be a fatal stroke.

## Case presentation

An 87-year-old man was conveyed to the emergency department after being found to be drowsy by his helper. He was watching television before he was found slumped in a chair, staring into space with saliva drooling. There was no history of recent fever, headache, fall, or trauma, and no recent hospitalization. Past medical history was significant for hypertension, stage 5 chronic kidney disease, a cerebrovascular accident, pacemaker insertion for sick sinus syndrome, a transurethral resection of the prostate for benign prostatic hyperplasia and pulmonary tuberculosis. His long-term medications were aspirin, omeprazole, amlodipine, and furosemide.

**Figure 1:**
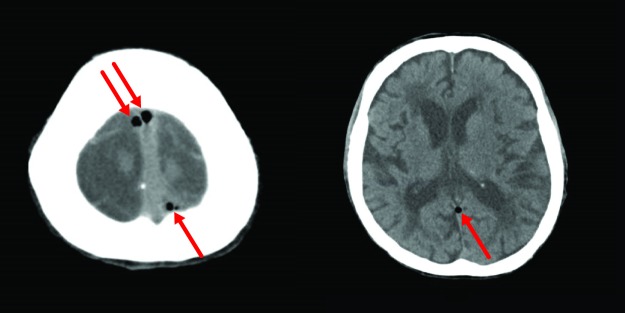
Initial CT Brain Initial computed tomography (CT) brain showing air within the superior sagittal (left) and straight sinuses (right)

On examination, his Glasgow coma scale was 7 (M4E2V1), and his pupils were equal and reactive. His vital signs were stable, with a temperature of 36.7 degree Celsius, pulse rate of 66 beats per minute, respiratory rate of 18 breaths per minute, oxygen saturation of 99% on room air, and blood pressure of 144/84 mmHg. He had a new onset right hemiparesis and normal reflexes. There was no evidence of any head injury or cerebrospinal fluid otorrhea or rhinorrhea. Stat capillary blood glucose was 8.7 mmol/L. Infective markers were normal, white blood count was 5.6 x103/µL, C-reactive protein was 0.3 mg/L, urea was 17.7 mmol/L (stable), sodium was 137 mmol/L, potassium was 5.1 mmol/L, creatinine was 331 µmol/L (stable), and corrected calcium was 2.29 mmol/L. The liver function test was normal. Electrocardiogram (ECG) did not show any evidence of acute myocardial ischemia. An urgent CT brain revealed air bubbles within the dural venous sinuses (bilateral cavernous, superior sagittal, straight, and left sigmoid sinuses) (Figure 1). There was no definite evidence of ischemic changes on CT brain. Pneumocephalus was initially thought to be the cause of his drowsiness and right hemiparesis. The neurosurgery team suggested conservative management of the pneumocephalus.

**Figure 2:**
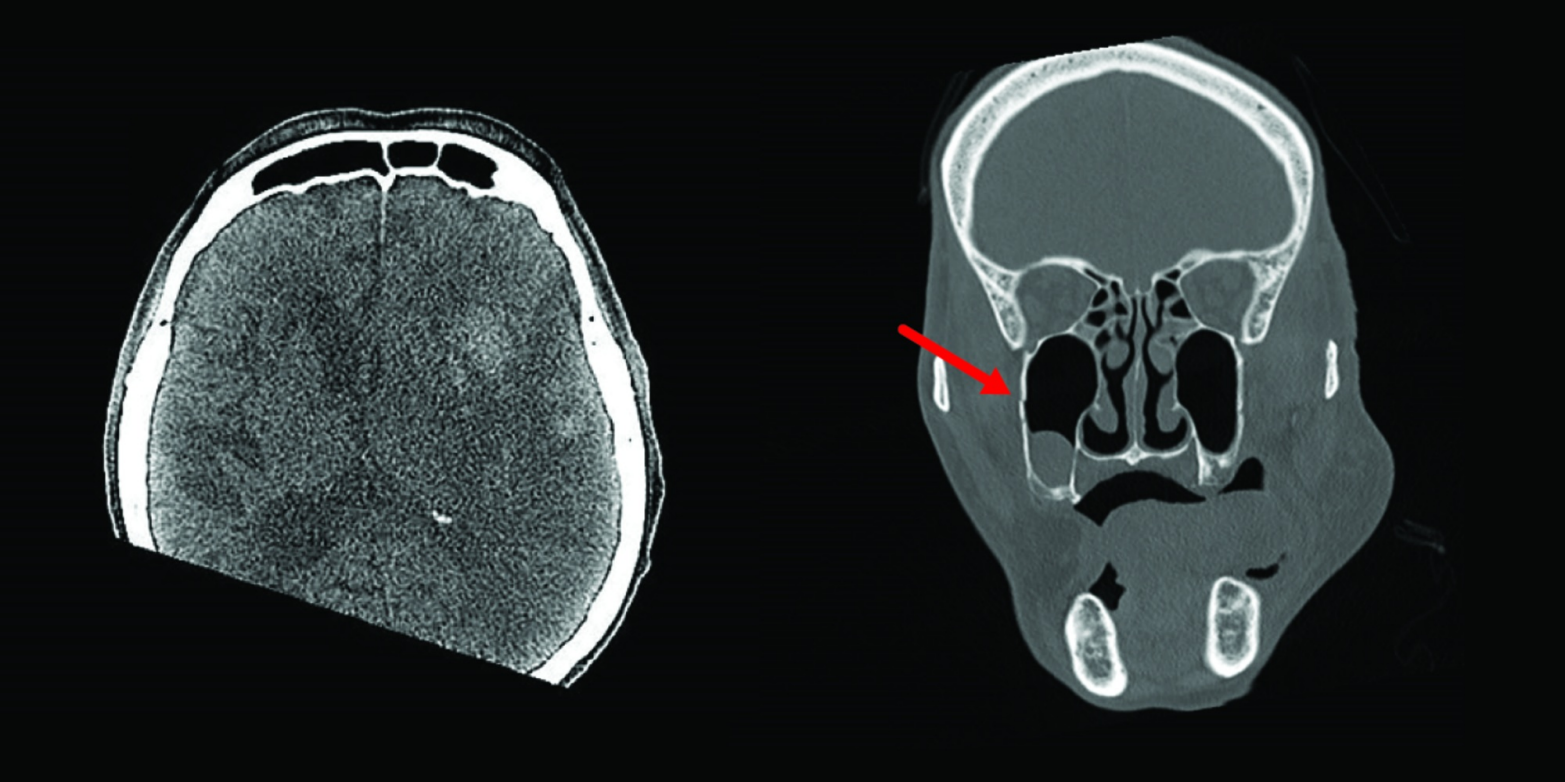
CT Facial Bone Computed tomography (CT) facial bone showing a left middle cerebral artery territory infarct with hemorrhagic conversion and midline shift (left) and fracture of the lateral wall of maxillary sinus (right)

A facial bone CT was ordered to rule out trauma, as it is the commonest cause of pneumocephalus. The CT scan, done two days later, demonstrated a minimally displaced fracture of the lateral wall of the right maxillary sinus. Of note, there was an acute left middle cerebral artery territory infarct with hemorrhagic conversion, a mass effect on the left ventricle, and a resultant midline shift (Figure 2). The pneumocephalus had resolved.

In view of a poor premorbid state, the severity of the stroke, and a lapse in the diagnosis of stroke, the patient was conservatively managed. He was started on statins; antiplatelets were held off in view of significant hemorrhagic conversion causing the midline shift. He underwent physiotherapy but remained bed-ridden.

A repeat CT brain one month later showed improvement in hemorrhagic transformation and a resolution of midline shift. He was then started on dual antiplatelet therapy. Despite the medical management, he demised one month after the initial presentation.

## Discussion

Pneumocephalus is an uncommon finding, even in patients with head trauma [2]. Other causes include cranial surgery, infection, and intracranial neoplasm [3]. Headaches, nausea, vomiting, seizure, or dizziness are common presentations. Although rare, subtle weakness, reflex abnormalities or frank hemiparesis may occur with focal parenchymal involvement. A fracture of the facial bone causes localized pneumocephalus; such a fracture of the lateral wall of the right maxillary sinus is not the cause of pneumocephalus in this case. Tension pneumocephalus is a clinical emergency where pneumocephalus produces a mass effect on the brain. In view of the lack of clinical features and the pathologic cause of pneumocephalus, combined with the extent and distribution of air (TY1), the cause of pneumocephalus in this patient was deemed idiopathic (TY2). 

Inadvertent intravenous injection of air via a peripheral catheter is presumed to be the cause in as high as 5% of all cases of pneumocephalus [1]. Pneumocephalus is usually conservatively managed. This includes head elevation and avoidance of coughing, sneezing, nose blowing, or the Valsalva maneuver. Definitive surgical treatment is indicated for symptomatic pneumocephalus or persistent pneumocephalus lasting more than one week.

**Figure 3:**
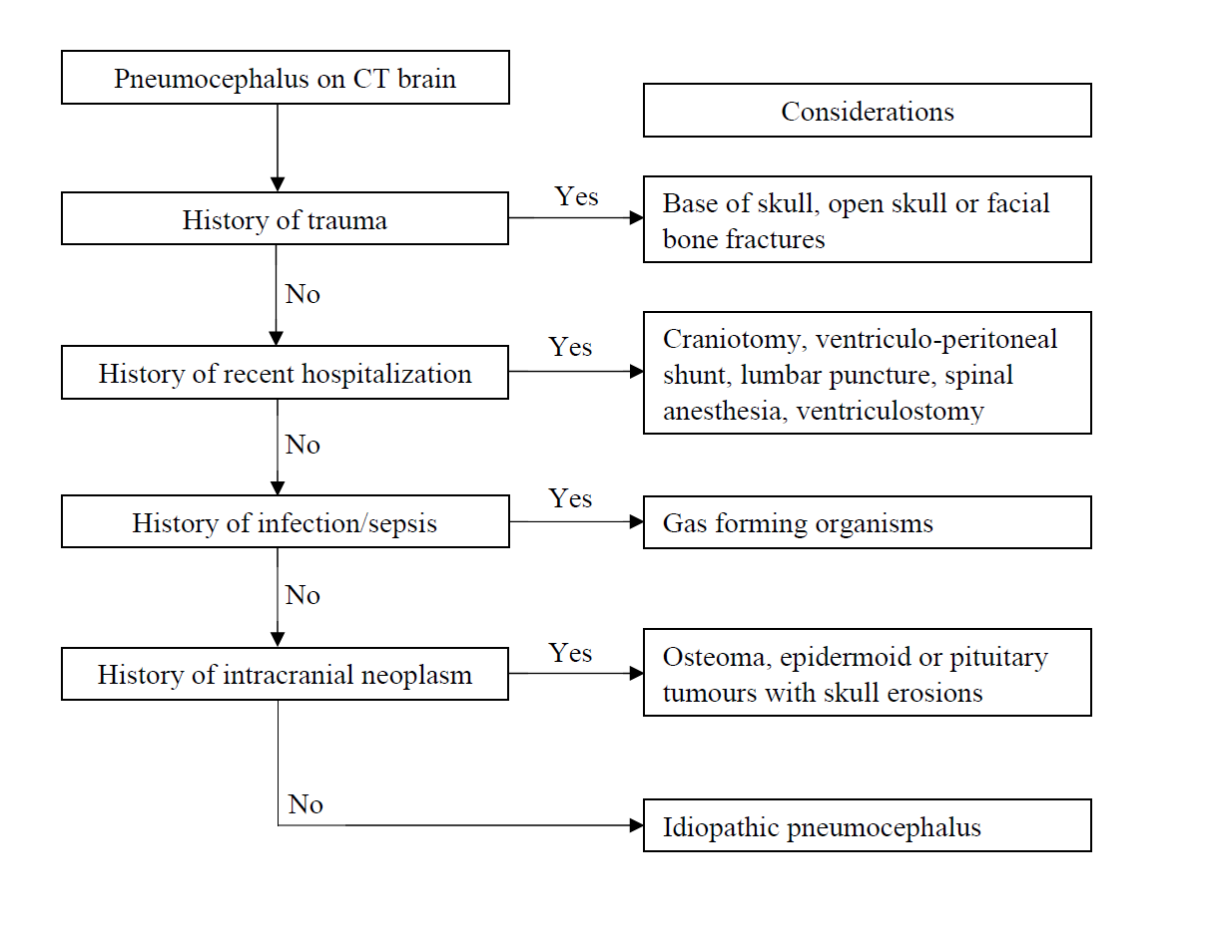
Diagnostic Workflow Diagnostic flowchart of pneumocephalus on brain imaging

We suggest a diagnostic flowchart in patients who are diagnosed with pneumocephalus on CT brain (Figure 3).  

In this patient, the change in mental status and right hemiparesis was attributed to pneumocephalus prematurely. It is uncommon for pneumocephalus to cause such changes. The sudden onset of drowsiness and new right hemiparesis should prompt a re-evaluation of the patient. A more detailed examination should be performed, particularly looking for gaze preference, hemispatial neglect, and Babinski sign. If the signs were present, a repeat CT or magnetic resonance imaging (MRI) brain could be done and definitive management could be planned earlier. In retrospect, the cognitive biases of premature closure and search satisficing were apparent.

The patient’s initial presentation was caused by an acute left middle cerebral artery territory infarct. Intravenous thrombolytic therapy should be considered in patients presenting less than 4.5 hours from symptom onset [4-5]. Mechanical thrombectomy is indicated for patients with acute ischemic stroke due to a large artery occlusion in the anterior circulation within six hours of symptom onset [6-7]. Despite a delay in the diagnosis, a poor premorbid state and the severity of the stroke, in this case, would preclude thrombolytic therapy or mechanical thrombectomy.

## Conclusions

Premature closure should be avoided; an alternative diagnosis ought to be pursued if the clinical presentation cannot be explained by the mere presence of gas in the cranium. In this case, this was especially true since drowsiness is an uncommon presentation of pneumocephalus and the distribution of gas was not in the territory that corresponded to the patient’s neurological deficit.
